# Proteomics Analysis of SsNsd1-Mediated Compound Appressoria Formation in *Sclerotinia sclerotiorum*

**DOI:** 10.3390/ijms19102946

**Published:** 2018-09-27

**Authors:** Jingtao Li, Xianghui Zhang, Le Li, Jinliang Liu, Yanhua Zhang, Hongyu Pan

**Affiliations:** 1 College of Plant Science, Jilin University, Changchun 130062, China; lijingtao789@126.com (J.L.); zhangxianghui@jlu.edu.cn (X.Z.); lile19890904@126.com (L.L.); jlliu@jlu.edu.cn (J.L.); 2 College of Plant Health and Medicine, Qingdao Agricultural University, Qingdao 266109, China

**Keywords:** *Sclerotinia sclerotiorum*, SsNsd1, compound appressorium, two-dimensional electrophoresis, proteomics analysis, differential expression proteins

## Abstract

*Sclerotinia sclerotiorum* (Lib.) de Bary is a devastating necrotrophic fungal pathogen attacking a broad range of agricultural crops. In this study, although the transcript accumulation of SsNsd1, a GATA-type IVb transcription factor, was much lower during the vegetative hyphae stage, its mutants completely abolished the development of compound appressoria. To further elucidate how SsNsd1 influenced the appressorium formation, we conducted proteomics-based analysis of the wild-type and Δ*SsNsd1* mutant by two-dimensional electrophoresis (2-DE). A total number of 43 differentially expressed proteins (≥3-fold change) were observed. Of them, 77% were downregulated, whereas 14% were upregulated. Four protein spots fully disappeared in the mutants. Further, we evaluated these protein sequences by mass spectrometry analysis of the peptide mass and obtained functionally annotated 40 proteins, among which only 17 proteins (38%) were identified to have known functions including energy production, metabolism, protein fate, stress response, cellular organization, and cell growth and division. However, the remaining 23 proteins (56%) were characterized as hypothetical proteins among which four proteins (17%) were predicted to contain the signal peptides. In conclusion, the differentially expressed proteins identified in this study shed light on the Δ*SsNsd1* mutant-mediated appressorium deficiency and can be used in future investigations to better understand the signaling mechanisms of SsNsd1 in *S. sclerotiorum*.

## 1. Introduction

*Sclerotinia sclerotiorum* (Lib.) de Bary is a destructive and hard-to-control plant necrotrophic fungal pathogen on a broad range of agricultural crops [1,2]. Developmentally, vegetative hyphae gathered together forming hardened, multicellular sclerotia enclosed by a melanized rind layer, which plays an important role in the development and pathogenesis of *S. sclerotiorum* [3,4]. Under suitable environmental conditions, sclerotia germinate to form vegetative hyphae or apothecia, and the latter release numerous ascospores that initiate new disease cycles [5]. Mycelia from sclerotia or ascospores can directly infect the plant tissues by forming compound appressoria (also known as infection cushions) from modified hyphae [2,4] or enter the plant tissue through open stomata by secreting oxalic acid [6]. Therefore, a better understanding of the developmental mechanism of appressorium is also critical to the control of this important plant disease.

The formation of compound appressoria in *S. sclerotiorum* has been reported to require a contact stimulus [7]. Prior to penetration, the tips of hyphae become swollen and extensively branched, and then form modified, multicellular, and melanin-rich compound appressoria [8,9]. The tip of compound appressorium could penetrate the host epidermis and form vesicles of bulbous [8]. Some events are consistent with this development process, such as the production and accumulation of oxalic acid (OA), cell wall-degrading enzymes (CWDE), and effector proteins, which contribute to *S. sclerotiorum* pathogenesis in myriad ways [9,10,11,12,13,14]. However, despite these important findings, the detailed molecular mechanism underpinning the development and formation of compound appressoria in *S. sclerotiorum* is still largely unclear.

In the past years, many genetic factors have already been characterized to be essential for appressoria development in *S. sclerotiorum*. The disruption of the oxalic acid biosynthesis gene (*Ssoah1*) promotes the compound appressorium development; however, the disruption of Sspks13 only eliminates the pigmentation of the compound appressorium without attenuating its infection and pathogenicity potential [2]. Significant accumulation of the oxalate decarboxylase (OxDC) gene *Ss-odc2* occurs during the compound appressorium development, and Δ*Ss-odc2* mutants were found to have less effective compound appressorium differentiation [11]. In addition, the secretory proteins Ss-Rhs1 and Ss-Caf1 were highly expressed during the hyphal infection process, whereas the silenced strains had decreased appressoria formation [9,15]. Furthermore, *Ss-ggt1* (γ-glutamyl transpeptidase gene), *sac1* (cAMP pathway adenylatecyclase gene), and *rgb1* (type-2A phosphoprotein phosphatase (PP2A) B regulatory subunit gene) have also been identified to be associated with the development of compound appressoria [16,17,18]. Recently, the type IV GATA zinc finger transcription factor SsNsd1, orthologous to the *Aspergillus nidulans* NsdD (never in sexual development) proteins and *Botrytis cinerea* BcLTF1 [19], was reported to regulate asexual–sexual development and appressoria formation [4]. Its knockout mutants were defective in the transition from hyphae to compound appressorium formation, resulting in a loss of infection-dependent pathogenicity on healthy hosts [4]. However, the signal pathway by which the SsNsd1 regulates the development and pathogenicity remains to be further elucidated.

Life sciences have been deeply influenced by the “omics” technologies in last decade, including genomics, transcriptomics, proteomics, and metabolomics, aiming at a global perspective on biological systems [20]. Proteomics strategies, such as the two-dimensional gel electrophoresis (2-DE) approaches, have been confirmed as efficient, rapid, and powerful means to identify proteins (or genes) followed by mass spectrometry, and matrix-assisted laser desorption/ionization (MALDI) [21]. Large-scale analyses of proteins by 2-DE have been conducted in a number of organisms, such as animals [22], plants [23], yeast [24], and fungi [25,26], which contributes considerably to our understanding of gene functions in the postgenomic era. However, the development and application of such methods in the filamentous plant-pathogenic fungus *S. sclerotiorum* have not yet been reported.

Modern agriculture faces a huge challenge in the prevention from the diseases caused by *S. sclerotiorum*. The transcription factor SsNsd1 was characterized to be essential for appressoria development in our previous study [4]. The possibility to control plant diseases by suppressing the compound appressorium formation would eliminate initial infections. Here, we used the SsNsd1 knockout mutant (Δ*SsNsd1*) to confirm the loss-of-function nature in compound appressorium development and conducted proteomics analysis by 2-DE. Using comparative proteomics analysis of the Δ*SsNsd1* mutant and the wild-type *S. sclerotiorum*, we attempted to identify the Δ*SsNsd1-*mediated differentially expressed proteins combined with peptide mass spectrometry analysis, which would contribute significantly to the SsNsd1-mediated compound appressorium formation. 

## 2. Results

### 2.1. Phylogenetic Analysis of SsNsd1 and Other GATA-Type Proteins

**Figure 1:**
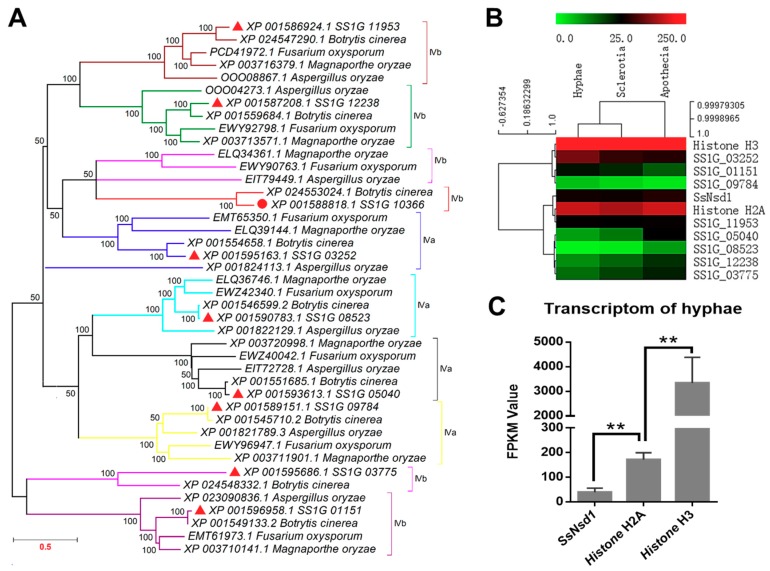
Phylogenetic analysis and transcription expression of SsNsd1 and other proteins containing GATA-type DNA domains in *S. sclerotiorum*. (**A**) Phylogenetic analysis of the amino acid sequences of SsNsd1 (SS1G_10366) and other GATA-type proteins in pathogenic fungi (*S. sclerotiorum*, *B. cinerea*, *F. oxysporum*, *M. oryzae*, and *A. oryzae*). A phylogenetic tree was generated by MAGE using the neighbor-joining method. The nine GATA-type proteins were separated by using a different branch color. (**B**) Hierarchical cluster of GATA-type genes and two histone genes in transcript abundance from three developments stages (hyphae, sclerotia, and apothecia) of *S. sclerotiorum*. Each gene is represented by a single row of colored boxes, and a single column indicates different development stages. The gene transcription abundance was evaluated by the fragments per kilobase of exon per million mapped fragments (FPKM) value. (**C**) The transcription level of *SsNsd1*, *Histone H2A*, and *Histone H3* genes at the hyphae developmental stage. The expression level of *SsNsd1* gene was significantly different from those of the histone genes (*n* = 3; ** *p* < 0.01).

In this study, similar proteins of SsNsd1 and other GATA-type proteins of *S. sclerotiorum* were searched from *Botrytis cinerea*, *Fusarium oxysporum*, *Magnaporthe oryzae*, and *Aspergillus oryzae* by BLAST. The homolog of SsNsd1 was obtained only from the *B. cinerea*. However, all other GATA-type proteins had homologs in *B. cinerea*, *F. oxysporum*, *M. oryzae*, and *A. oryzae*. Phylogenetic analysis of the putative amino acid sequence of these GATA-type proteins showed their genetic relationship in different fungi (Figure 1A). Coincidently, the GATA-type proteins of *S. sclerotiorum* were closely related to *B. cinerea*. Besides, two forms of the type IV zinc finger motif (IVa and IVb) are also depicted in Figure 1A based on the residue loops. Most of the clades contained all five sequences from *S. sclerotiorum*, *B. cinerea*, *F. oxysporum*, *M. oryzae*, and *A. oryzae*. However, the SS1G_03775 and its homologs were separated as shown by the pink color clade. One branch was also separated from the clade of the SS1G_03252 and its homologs, which might be due to the poor similarity in different fungi.

### 2.2. Transcript Accumulation of SsNsd1 and Other GATA-Type Proteins

Digital gene expression (DGE) analysis based on FPKM values was performed using the transcriptomes during three the developmental stages of *S. sclerotiorum* (Figure 1B). The transcript accumulation in the GATA-type proteins was lower than those of the histone genes (*histone H3* and *histone H2A*). Only the SS1G_03252 protein showed a little higher transcript accumulation in the hyphae development stage, whereas most of the other GATA-type proteins displayed low contents with FPKM values ranging from 1.3 to 64 in all three development stages. Obviously, the SS1G_08523 and SS1G_09784 proteins were extremely low abundant with FPKM values under 10 in all three development stages.

As one of the GATA-type proteins, the varied expression patterns of SsNsd1 were previously examined and compared across developmental stages [4]. In this study, to further determine the regulation mechanism of the Ssnsd1 expression, we obtained its expression profile in the vegetative hyphae development stage (Figure 1C). The transcript accumulation of the *SsNsd1* gene was significantly lower than those of the histone genes (*histone H2A* or *histone H3*) during the vegetative mycelial growth prior to the compound appressorium formation. *SsNsd1* was expressed at the lowest level (FPKM value of 39.6), however, *Histone H2A* was expressed higher (FPKM value of 171.8). Moreover, *Histone H3* (FPKM value of 3337.3) displayed the highest expression level, indicating its predominant role, usually as a housekeeping gene. Overall, the transcript accumulation of SsNsd1 and other GATA-type proteins were exceedingly low in the development stages of *S. sclerotiorum*.

### 2.3. ΔSsNsd1 Mutant Suppressed Compound Appressorium Formation

**Figure 2:**
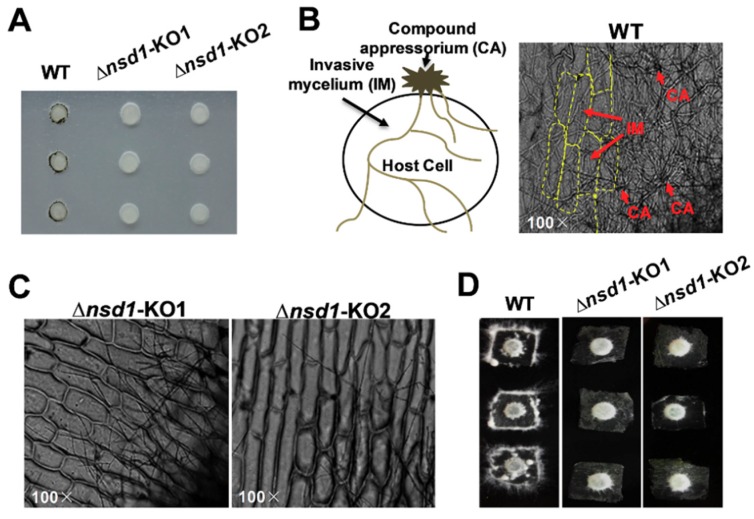
The defective compound appressoria of Δ*SsNsd1* mutant strain led to a loss of penetration into unwounded onion tissue. (**A**) Pigmented compound appressoria of wild type (WT) were observed on parafilm. Pictures were taken four days after transfer (DAT) of 5-mm-diameter mycelial plugs to parafilm. (**B**) Penetration assays with the WT on onion epidermal strips. Invasion mycelium (penetration) of WT strain on onion epidermal strips was observed by light microscopy two days after inoculation (DAI). (**C**) Penetration assays with the Δ*SsNsd1* strain on onion epidermal strips at 2 DAI. No invasion mycelium was observed on onion epidermal strips inoculated by Δ*SsNsd1* strain. (**D**) Penetration assays with the WT and the Δ*SsNsd1* mutant on onion epidermal strips at 4 DAI.

Although SsNsd1 exhibited a low expression level during the hyphae development, it still played a crucial role in appressorium development (Figure 2). Phenotypically, the Δ*SsNsd1* mutant had inhibited normal production of pigmented compound appressoria from the vegetative hyphae, as established by paraffin film assays (Figure 2A). In determining whether Δ*SsNsd1* affected the compound appressorium formation or only the pigmentation, normal compound appressorium and penetration (invasive mycelium) were observed microscopically only on onion epidermal strips inoculated with the wild type (WT) strain (Figure 2B), but not on the Δ*SsNsd1* mutant strain. The WT strain could colonize onion cells, but no invasive mycelium was observed from Δ*SsNsd1* mutants (Figure 2C). Thus, SsNsd1 abolished the compound appressoria formation from the modified hyphae, resulting in the penetration-dependent loss of pathogenicity.

### 2.4. Diagram of the Identification of Differential Proteins

**Figure 3:**
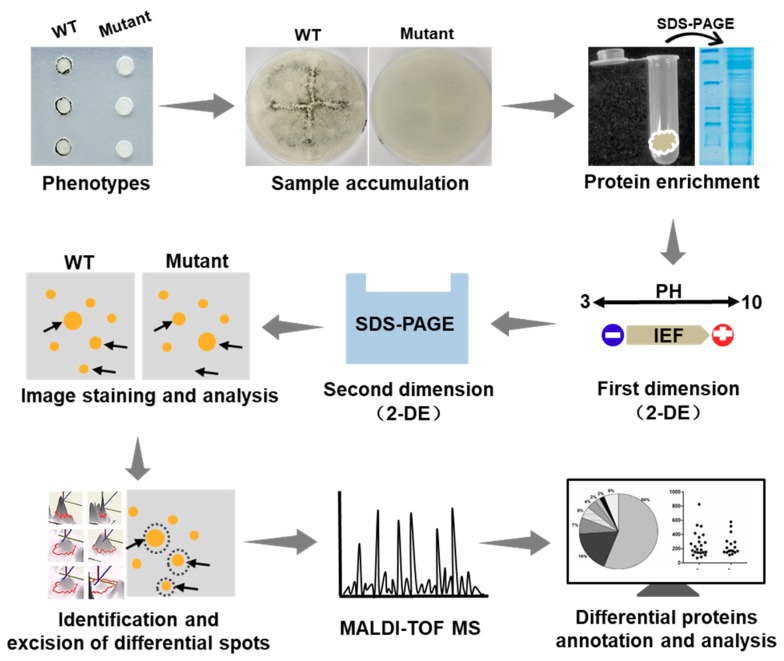
Schematic diagram illustrating the process of identifying the changes of proteomics between the Δ*SsNsd1* mutant and wild type (WT) during the compound appressorium formation. Generally, tissue samples with different phenotypes were subjected to protein extraction and SDS-PAGE test. Then the proteomics profiles were analyzed by two-dimensional gel electrophoresis (2-DE) to obtain the differential expression spots, which were further identified by mass spectrometry (MS) of the peptides and bioinformatics analyses (2-DE, two-dimensional gel electrophoresis; IEF, isoelectric focusing; SDS-PAGE, sodium dodecyl sulfate polyacrylamide gel electrophoresis).

In this study on *S. sclerotiorum*, we applied the 2-DE technology. A diagram illustrating the approach of the determination of the proteomics changes is presented in Figure 3. In this diagram, the comprehensive protocol is described by individual steps of the application of this technique, i.e., sample preparation and solubilization, isoelectric focusing (IEF) in IPG strips, running SDS-PAGE gels, image analysis, differential spots identification and mass spectrometry (MS) analysis, and bioinformatic prediction. 

### 2.5. Protein Extraction from Enriched Compound Appressoria

**Figure 4:**
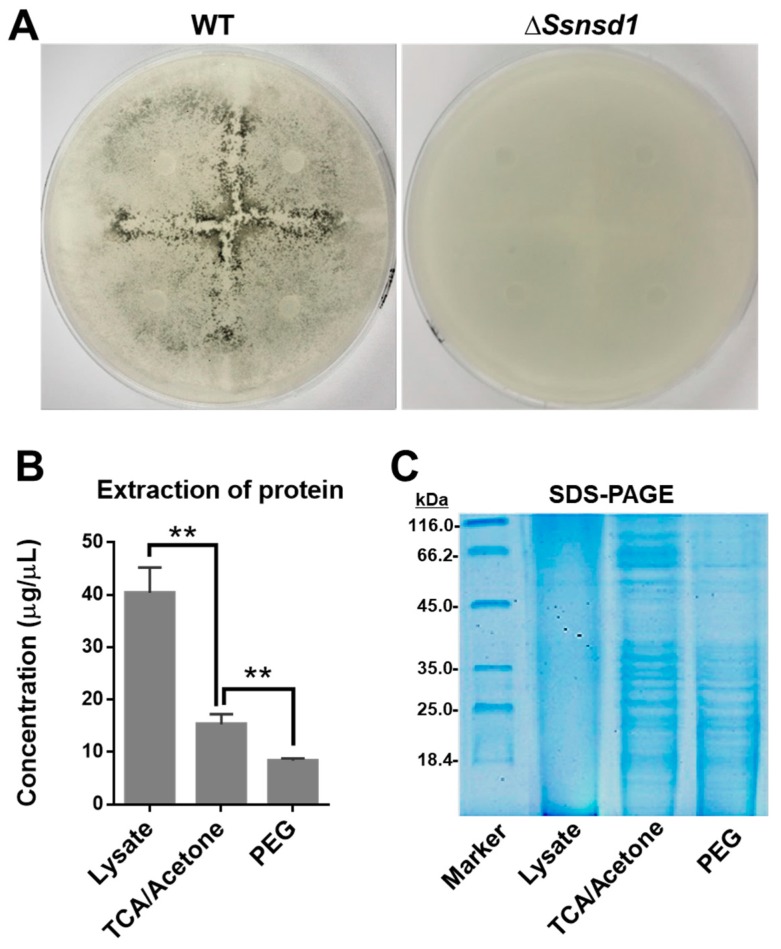
Protein extraction and SDS-PAGE test of accumulated compound appressoria tissue induced on cellophane. (**A**) Induction assay for compound appressoria development on potato dextrose agar (PDA) overlaid with cellophane at 3 DAI. (**B**) Quantification of protein concentration, which was enriched by three different extraction methods: lysate, trichloroacetic acid (TCA)/acetone precipitation, and polyethylene glycol PEG method (*n* = 3; ** *p* < 0.01). (**C**) Quality detection of proteomics by SDS-PAGE to optimize the method for protein extraction.

The cellophane induced the formation of abundant compound appressoria from the modified hyphae, which were observed macroscopically when the WT was cultured on cellophane (Figure 4A). No pigmentation was observed due to the lack of compound appressoria when Δ*SsNsd1* were cultured on cellophane (Figure 4A). Thus, this was an effective method to provide enriched compound appressoria tissue with simple sample collection, which was ideally suited for protein extraction.

**Figure 5:**
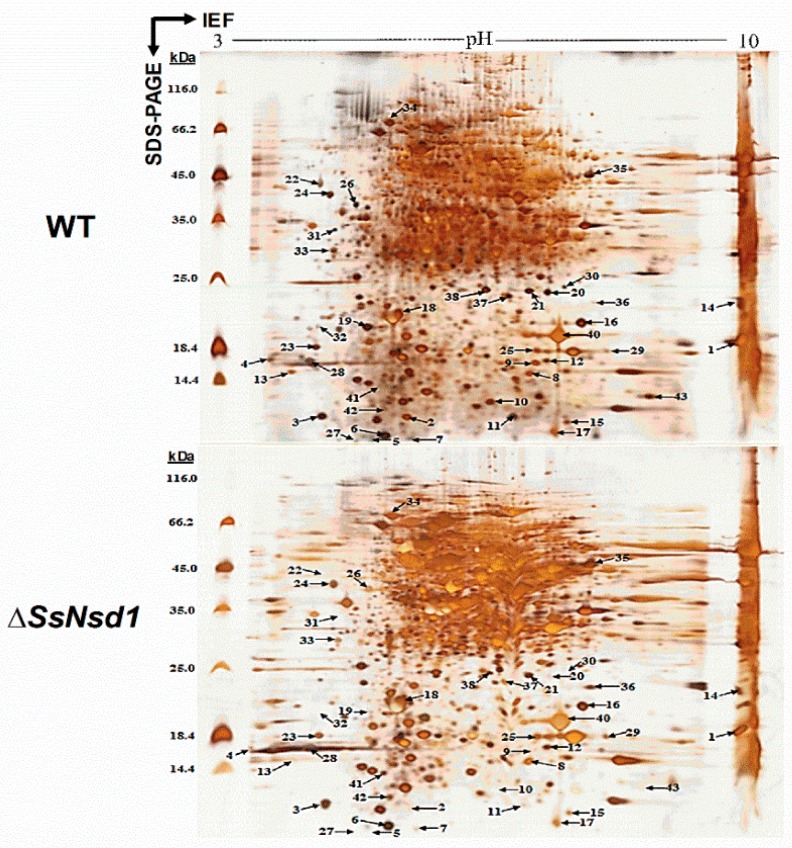
Silver nitrate-stained 2-DE gel image of the WT and Δ*SsNsd1* strains. The proteins were extracted from the accumulated compound appressoria tissue induced by cellophane. The numbers and arrows correspond to the identified differential expression proteins (≥3-fold change) for further MS analysis of the peptides.

To optimize the best protein extraction method for 2-DE separation, we compared the concentration and quality of the extracted proteins using the three different methods. The highest concentration of protein was extracted by the use of the lysate as compared with those extracted by trichloroacetic acid (TCA)/acetone or polyethylene glycol (PEG) precipitation (Figure 4B); however, the quality of this protein, established by the SDS-PAGE test, was extremely poor (Figure 4C). In contrast, the protein extracted by further TCA/acetone or PEG precipitation exhibited clearer bands, but that extracted by TCA/acetone precipitation was at a higher concentration; the best result was obtained using the SDS-PAGE test (Figure 4C). Furthermore, the 2-DE clearly showed protein spots aggregation and transparent background (Figure 5). Thus, the protein extraction with further TCA-acetone precipitation could lay a foundation for a novel approach in comparative proteomics analysis by 2-DE technology in *S. sclerotiorum*.

### 2.6. Identification of the Differential Protein Spots by 2-DE Analysis

**Figure 6:**
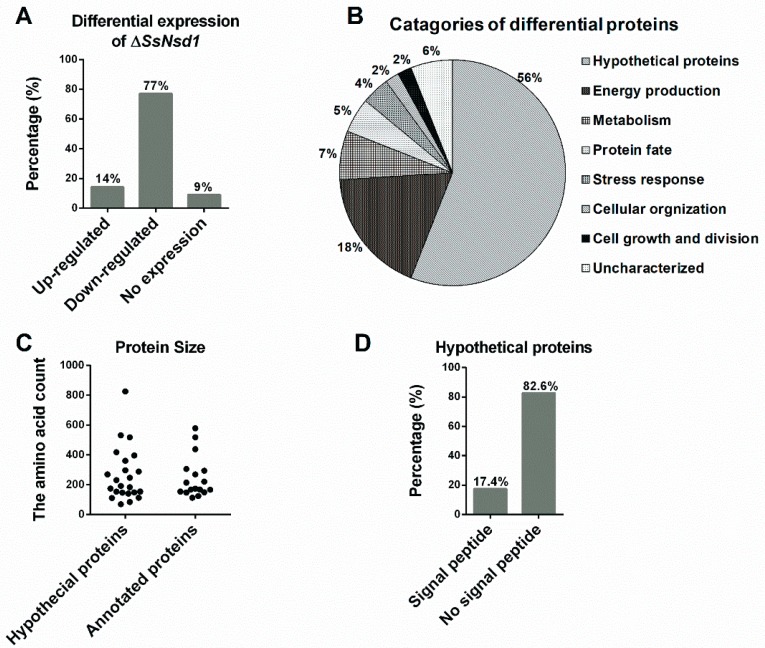
Analysis and evaluation of differentially expressed proteins. (**A**) Differential expression analysis of the identified protein spots in the Δ*SsNsd1* mutant compared to the WT strain. (**B**) Functional categories of differential expression proteins after MS identification and functional annotation. The percentage corresponds to the proportion of the annotated proteins in the classification. (**C**) The population distribution of the protein size evaluated by the amino acid count of the predicted proteins. (**D**) The signal peptide was predicted by running amino acid sequences of predicted hypothetical proteins on the SignalP server.

The proteins extracted from the WT and Δ*SsNsd1* culture on cellophane were separated by 2-DE (Figure 5). In this figure, 2-DE displayed well-visible protein spot aggregation and transparent background, while few nonspecific bands or foreign matters were observed. Using three biological replicates for both the wild-type and mutant strains, the clear 2-DE images were compared and analyzed by ImageMaster™ 2D Platinum 6.0 software. More than 2660 protein spots were detected reproducibly on each 2-DE gel image for the WT and Δ*SsNsd1* mutant, within the pH range of 4 to 10 and with relative molecular masses of 8 to 80 kDa. However, only 43 protein spots exhibited changes in the differential abundance (more than three-fold) between the WT and the Δ*SsNsd1* mutant, which are marked with arrows and numbers in Figure 5. Among these selected differential protein spots, 33 protein spots were downregulated, six proteins spots were upregulated, and four proteins spots disappeared in the Δ*SsNsd1* strain compared to the wild type (Figure 6A). Subsequently, all differential expression proteins were excised and subjected to MALDI-TOF analysis.

### 2.7. Prediction and Characteristics of the Identified Proteins

After prediction and functional annotation of these excised proteins, 40 proteins were identified. Of them, 17 proteins (38%) were predicted with the known functions (Table 1), and other 23 homologous to unnamed or predicted proteins were collectively designated as “hypothetical proteins”, accounting for 56% (Table 2). However, three proteins (spots 7, 13, and 23) were not identified by MALDI for unknown reason, which were designated as “uncharacterized” (6%). The predicted known functional categories (38%) were further sorted into six functional categories, including energy production (18%), metabolisms (7%), protein fate (5%), stress responses (4%), cellular organization (2%), and cell growth and division (2%) (Figure 6B). 

In addition to providing functional categories, the global view of the protein sizes was also evaluated by the numbers of the amino acids (Figure 6C). Based on the function annotation, they were divided into hypothetical and annotated proteins. Then, the distribution of their protein size was similar to that of 262 and 247 amino acids, respectively (Figure 6C). Furthermore, we predicted the signal peptide among these hypothetical proteins to obtain additional insights into the putative functions of the hypothetical proteins (Figure 6D, Table 2). Four proteins (17.4%) were predicted to contain the N-terminal signal peptide, which indicated they might be potential secretory proteins during the compound appressorium formation.

### 2.8. Functional Analysis of Annotated Proteins

The 2-DE approach provided a powerful proteomic screening tool to identify the initial candidate differentially expressed proteins. We evaluated these predicted proteins after functional annotation and identified 17 proteins to be functionally known, representing 10 nonredundant unique proteins (Table 1). The fact that identical proteins were available in different protein spots might have been due to the protein modification or other unclear reasons as analyzed in the discussion section.

Here, we provide information concerning the analysis of the predicted functionally known proteins. Nucleoside diphosphate kinase (NDPK) (spots 1, 9, and 17) usually possesses kinase activity exerted by direct response to the G-protein signaling or indirect catalytic GDP–GTP exchange activity, which also plays a major role in the synthesis of nucleoside triphosphates [27,28,29]. The eukaryotic translation initiation factor 5A-1 (eIF5A-1; spot 18) is involved in the protein fate pathway [30], which activates the 60 s subunits combination, assists in the conformational changes of the 80 s subunits, and participates in the intracellular part; proteins associate with ribosomes cyclically during the elongation phase of the protein synthesis [31]. The elongation factor 1-β (spot 26) plays a central role in the elongation step in eukaryotic protein biosynthesis [32]. The 60 s ribosomal protein L23 (Spot 4) is usually involved in cell growth; its expression was decreased in the mutant (Table 1). Ribosomal protein is involved in regulating gene transcription, translation, and regulation of cell proliferation, differentiation, apoptosis, etc. [33].

The nuclear transport factor 2 (spot 3) mediates the nucleus introduction of GDP-bound RAN (ras-related nuclear) from the cytoplasm, which is of great significance in the cargo receptor-mediated nucleocytoplasmic transport [34]. The GTP-binding nuclear protein (spot 30) is also known as the GDP-bound RAN, which is involved in the nucleocytoplasmic transport processes, nuclear envelope formation, and mitotic spindle formation [35].

The predicted ubiquitin-conjugating (UBC) enzyme E2 (spot 8) belongs to the ubiquitin pathway enzymes, which are involved in protein degradation in eukaryotic cells [36]. The SCF (Skp1/Cul1/F-box) complex submit Skp1 (spot 32) is involved in the assembly of protein complex and joins in ubiquitin depending on the protein catabolism process [37]. Peptidyl-prolyl cis-trans isomerase (spots 12, 25, 16, and 39) was downregulated, whereas spot 29 was upregulated (Table 1). Peptidyl-prolyl *cis*-*trans* isomerase regulates the mitosis-related protease in the cell cycle by protein phosphorylation of the substrate proteins [38] or through other mechanisms such as the ubiquitin-mediated proteasomal degradation [39].

Citrate synthase (spot 35) is localized in the mitochondrial matrix and catalyzes the condensation reaction from acetyl coenzyme A (CoA) and oxaloacetate to form the six-carbon citrate [40]. Oxalic acid biogenesis is realized through the hydrolysis of oxaloacetate, which is a key pathogenicity factor accumulated during the compound appressorium development [10].

The functional analysis of the predicted differential proteins was mainly based on the evidence in mammalian, plant, or yeast cells. However, the proteins displayed their important role on the cell proliferation, differentiation, protein synthesis and degradation, protein transport and modification, etc., which might determine they function as a complex regulatory network during the compound appressorium formation in *S. sclerotiorum*.

## 3. Discussion

Novel strategies for prevention and control of the devastating plant pathogenic fungus *S. sclerotiorum* have been intensively investigated [5]. However, still no effective method has been discovered to control the diseases caused by this pathogen. Compound appressoria are formed unless penetration occurs directly via stomata, which could be one of the key targets for disease control. The GATA-family transcription factors are involved in several essential aspects of the life cycle of *M. oryzae*, especially in the regulation of appressorium development and sporulation [41]. In *S. sclerotiorum*, the GATA-type transcription factors SsSFH1 and SsNsd1 were recently reported to be involved in the development of compound appressoria [4,42]. Here, phylogenetic analysis was performed, and transcription accumulation of all the predicted GATA-type proteins was detected. Importantly, even the transcription accumulation of *SsNsd1* in the vegetative hyphae was significantly higher than that during the sclerotium and apothecium developmental stages [4]. The transcript accumulation remained at a much lower level even during vegetative mycelial growth, compared to that of the histone genes (Figure 1C). The *SsNsd1* gene knockout strain was defective in the development of appressorium, and no penetration was observed into unwounded onion epidermal cells (Figure 2), which confirmed the findings of a previous study [4]. In general, NsdD or its orthologous gene is involved in providing a regulatory balance between asexual and sexual development in ascomycete fungi (e.g., the development of perithecia, fruiting body, conidia, or sclerotia) [43,44,45]. In addition, NsdD is also involved in pathogenicity. The Δ*bclft1* mutants of *B. cinerea* exhibited only a postpenetration virulence defect without causing significant defects in the compound appressorium development; however, the *S. sclerotiorum* Δ*Ssnsd1* mutant was essentially reversed as established earlier [4]. Due to the particularly different infection defects between *B. cinerea* and *S. sclerotiorum*, in the present study, we focused on the compound appressorium deficiency phenotype and the biological role of SsNsd1, aiming to find the key target of appressorium formation-related genes that would enable the control of this pathogen.

The proteomics analysis as an evaluation of the final level of gene expression started out with techniques based on 2-DE and extended its reach by the use of MS-based techniques that have been increasingly employed in recent years [20]. Although alternative technologies, such as multidimensional protein identification technology (MudPIT), or arrays, have already emerged, thus far, there is no technology that matches 2-DE in its capability to realize routine parallel expression profiling of large mixtures [46]. Furthermore, 2-DE combined with the identification by MS is currently the major approach utilized in most of the undergoing proteome projects to develop a global understanding of the living cell [46]. Compared to the quantitative analysis based on MALDI-TOF techniques, LC–MS is currently in an early stage considering limitations, such as the availability of software, algorithms, etc. [20]. MALDI-TOF is already widely used in fungal proteome research, such as that in yeast [24], *A. fumigatus* [25], and *Cryomyces antarcticus* [26]. Therefore, we applied the 2-DE technology and MALDI-TOF mass spectrometry in this project to investigate the plant pathogenic fungus *S. sclerotiorum* using proteomics analysis for identification of differentially expressed proteins during the compound appressorium formation (Figure 3). Using suitable equipment and experienced laboratory personnel, this system approach can quickly perform the identification of functional proteins.

The 2-DE technology combined with IPGs has already conquered most limitations of carrier ampholyte-based 2-DE with in the respect of reproducibility, handling, resolution, and separation [47]. The efforts to develop the 2-DE technology further have been concentrated on improved solubilization/separation of hydrophobic proteins, show of low abundance, and more reliable quantitation by fluorescent dye technologies in recent years [46]. Despite the obvious advantages of the 2-DE technology, high quality proteins samples are always the bottleneck and precondition to the 2-DE project approach. As the 2-DE was firstly applied in *S. sclerotiorum*, we provided an optimal method for sample preparation after comparing three different pathways for protein extraction (Figure 4). By SDS-PAGE test, the protein extracted by further TCA/acetone precipitation and PEG methods has a better quality and could be used for 2-DE analysis. Both TCA/acetone and PEG methods are useful for minimizing protein degradation and removing interfering compounds, such as salt or polyphenols [48]. However, the amount of protein extracted by the PEG method is difficult to meet the requirements of 2-DE as established based on our results and those of a previous study [49]. In a comprehensive analysis, the TCA/acetone method displayed its advantages and was found to be the best method for protein extraction, which was consistent with the findings of a previous examination on another fungus, *A. fumigatus* [50]. Furthermore, in the 2-DE analysis, we achieved the separation of as many protein spots as needed on the gel, which was a prerequisite for the computerized analysis (Figure 5). In addition, a clear peptide mass spectrum was finally obtained by extraction of the different expressed protein spots, which laid the foundation of further research on SsNsd1-mediated differentially expressed proteins. 

The development of the compound appressoria involves several distinct stages [9] and is tightly regulated by numerous genetic factors. In the present study, a total of 43 differentially expressed proteins were identified with significantly differential expression changes (≥3-fold) by computer analysis (Figure 5 and Figure 6). Most of them were downregulated, which indicated that the SsNsd1 transcript factor might positively regulate them. SsNsd1 might exert a reverse role in the signal pathway of the upregulated protein spots. By MS analysis of peptides and functional annotation, these functionally known proteins were predicted to be involved into energy production, metabolism, protein fate, stress response, cellular organization, and cell growth and division. However, attention had to be paid to the hypothetical proteins as they contained the signal peptide. The secretion and accumulation of effector proteins are usually coincident with the appressorium formation process, which contributes to *S. sclerotiorum* pathogenesis [9,15]. Therefore, these newly identified four proteins might have the effector protein role during the compound appressoria formation, but this notion needs to be further studied (Table 2). Overall, the differentially expressed proteins were finally obtained from the Δ*SsNsd1* mutant, which might play an important role during the compound appressorium formation. Furthermore, losing the capacity to produce compound appressorium could also lead to defective sclerotium development, which is a key factor in the disease cycle of *S. sclerotiorum*, such as the mutation of *Ss-ggt1* (a γ-glutamyltranspeptidase gene) [16], *sac1* (a cAMP pathway adenylatecyclase gene) [17], and *rgb1* (a type-2A phosphoprotein phosphatase (PP2A) B regulatory subunit gene) [18]. Therefore, these identified differential expression proteins were important gene resources involved in the development of *S. sclerotiorum*, which might be associated with the formation of both compound appressoria and sclerotia.

Last but not the least, a section on how to evaluate the 2-DE and the identified differential proteins is included here. After 2-DE, each protein could be theoretically resolved at a unique isoelectric point/molecular size coordinate [51]. Although hundreds of protein spots were also separated on the gel, omission of partial differential proteins can always occur due to unfavorable experimental factors, such as incomplete precipitation and/or dissolution of proteins [48], loss of sample during gel entry, inefficiency transfer of the protein from the first to the second dimension, loss of protein during staining [20], and truly absent spots from the samples [52]. In addition, some spots from 2-DE might result multiple protein identification, however, only the first identified protein with best protein score and most peptide counts was accepted for further study. Moreover, attention had also to be paid to the identical proteins (listed in Table 1), such as the nucleoside diphosphate kinase, peptidyl-prolyl cis-trans isomerase, and the 60 s ribosomal protein. Post-translational protein modifications affect the isoelectric point and, therefore, the focusing behavior of the protein in the first dimension [23], which could lead to the presence of identical proteins in different locations (i.e., spot 25 and spot 29; spot 4 and spot 28) (Table 1 and Figure 5). Post-translational modifications by fatty acid acylation, glycosylation, methylation, acetylation, or phosphorylation largely modulate the activity of most eukaryote proteins [53,54]. For example, certain signaling pathways were found to consist of series of phosphorylation and dephosphorylation events, which defined directionality and allowed different levels of feedback regulation [55]. In addition, the incomplete and insensitive separation can also lead to the appearance of identical proteins in different locations. Besides, attention is to be paid to the silver staining, as Coomassie brilliant blue was used in most of the 2-DE staining [56]. The silver staining has a low dynamic range, which has been criticized for the quantitative analyses of spots. However, the silver staining has very high sensitivity, which allows for a detection of very low protein amounts [20]; the improved and advanced image processing method could become feasible in better quantification of protein spots [57]. Besides, to obtain more data of the exact protein abundance, only the differential proteins with ≥3-fold changes were accepted for further study in this research. Therefore, the 2-DE technology of gradual optimization, further analysis of protein modifications, and other proteomic analysis methods are still needed to employ, which would present formidable challenges but generate indispensable insight into biological functions in *S. sclerotiorum*.

## 4. Materials and Methods

### 4.1. Fungal Strains and Culture Conditions

The wild-type (WT) *S. sclerotiorum* isolate 1980 and its derived mutant Δ*SsNsd1* were used in this study based on our previous reports [4]. The strains were routinely grown on potato dextrose agar (PDA) at normal room conditions. The WT and Δ*SsNsd1* stocks were stocked as dry sclerotia or as desiccated mycelia-colonized filter paper at −20 °C.

### 4.2. Phylogenetic Analysis of SsNsd1 and Other GATA-Type Proteins

From the genome of *S. sclerotiorum*, nine proteins are predicted to containing GATA-type DNA domains: SS1G_1036, SS1G_11953, SS1G_12238, SS1G_03252, SS1G_08523, SS1G_05040, SS1G_09784, SS1G_03775, and SS1G_01151 [4,42]. The BLASTX program at NCBI (http://www.ncbi.nlm.nih.gov/) was employed to search for the homologs of the sequence of the SsNsd1 (SS1G_1036) and other GATA-type proteins from pathogenic fungi (*Botrytis cinerea*, *Fusarium oxysporum*, *Magnaporthe oryzae*, and *Aspergillus oryzae*). The phylogenetic tree was generated using neighbor-joining method in MEGA5 [58]. Prediction of protein zinc finger domain was performed to classify the different categories. Most fungal GATA factors contain a single zinc finger domain, which can be divided into two distinct categories: the 17-residue loops (CX_2_CX_17_CX_2_C; zinc finger type IVa) and the 18-residue loops (CX_2_CX_18_CX_2_C; zinc finger type IVb) [59,60].

### 4.3. Digital Gene Expression of SsNsd1 and Other GATA-Type Proteins

The transcription accumulation of SsNsd1 from three different developmental stages (hyphae, sclerotia, and apothecia) has been characterized by qRT-PCR [4]. To further study the gene expression patterns of SsNsd1 prior to compound appressorium development, we profiled gene expression patterns in *S. sclerotiorum* from hyphae, sclerotia, and apothecia through RNA-seq approach. The transcription level of SsNsd1 and other GATA-type proteins were quantified using the fragments per kilobase of exon per million mapped fragments (FPKM) method [61]. The FPKM means were generated from three technical replicate samples. The FPKM values of *histone H3* (SS1G_09608.3) and *histone H2A* (SS1G_02052) were used as endogenous control for quantitative comparison with *SsNsd1* and other GATA-type protein genes. Hierarchical clustering was performed using the MeV program [62]. Clustering was based on the average of FPKM values.

### 4.4. Compound Appressorium Assays

Deficiency of compound appressoria was observed macroscopically by placing the freshly colonized mycelial agar plugs (5-mm diameter) on parafilm due to the presence of pigmented appressoria surrounding the agar plug [2]. Yellow onions were purchased from a local grocery store, and the onion epidermal strips were used for inoculation with a colonized PDA agar plug for observing the penetration of compound appressorium using light microscopy. For the enrichment of compound appressoria, colonized agar was cultured on PDA medium covered with cellophane and grown at a temperature of 22 to 25 °C as reported previously [2].

### 4.5. Two-Dimensional Gel Electrophoresis (2-DE) Strategy in This Study

The 2-DE combines isoelectric focusing separation based on isoelectric point of protein in the first dimension and sodium dodecyl sulfate (SDS) polyacrylamide gel electrophoresis (SDS-PAGE) to separate the complex mixtures of proteins according to the molecular size in the second dimension [23]. The Combined with identification by mass spectrometry (MS), 2-DE is currently the major method used in the majority of the ongoing proteome projects [46]. Besides, 2-DE gels are easy to handle and could be produced in a highly parallelized way. Furthermore, the corresponding software has also reached a level that allows for routine bioinformatic analysis. Meanwhile, in the presence of a suitable laboratory equipment and experienced personnel, analysis of samples can be theoretically completed through this approach with investments of time and efforts that are much smaller than those needed for the laboratory work [20]. Thus, these mature and coherent techniques are well-suited for comparative proteomics analysis of Δ*SsNsd1* mutant-mediated appressoria deficiency in *S. sclerotiorum*. In detail, tissues derived from two different strains, WT and Δ*SsNsd1*, were harvested, and the proteome was enriched and solubilized. The protein mixture was then applied to a “first dimension” gel strip that separated the proteins based on isoelectric focusing (IEF) in IPG strips. Next, the IPG strip was subjected to equilibration and running of multiple “second dimension” SDS–PAGE gels, where proteins were finally separated by their molecular charge and molecular size. After staining, the visual protein spots were recorded and analyzed by sophisticated software. Then, the differential protein spots were excised for MS analysis. Finally, the differential proteins were subjected to functional annotation and prediction analysis.

### 4.6. Protein Extraction and Optimization

The enriched compound appressoria on the cellophane were harvested and frozen in liquid nitrogen, and then ground to a fine powder for protein extraction. To optimize the method for protein extraction from *S. sclerotiorum* and separate the protein by 2-DE, during the compound appressoria production, the protein was extracted by direct lysate and further TCA/acetone or PEG precipitation methods. For the lysate method [63], 0.1 g powdered sample was suspended in 300 µL of precooled lysate (7 M urea, 2 M thiourea, 2% (*w*/*v*) 3-[(3-Cholamidopropyl)dimethylammonio]propanesulfonate (CHAPS), 20 mM Tris-HCl, and 20 mM dithiothreitol), then vortexed for 30 s and centrifuged at 15,000× *g* rpm for 10 min at 4 °C. The supernatant was further centrifuged at 15,000× *g* rpm for 30 min. Approximately 250 µL of the supernatant was taken as the crude protein solution and stored in a freezer at −80 °C. For further precipitation by trichloroacetic acid (TCA)/acetone [64], 80 µL of crude protein solution was suspended in 5 mL of cold TCA/acetone (10% TCA, 0.07% β-Mercaptoethanol (ME) in acetone) and mixed for 30 s, then precipitated at −20 °C for 2 h. Further, the precipitate was centrifuged at 15,000× *g* rpm for 15 min at 4 °C. Then, the supernatant was discarded, and the pellet washed three times with 800 µL of cold acetone and finally centrifuged at 15,000× *g* rpm for 15 min at 4 °C. Next, the pellet was desiccated using a vacuum dryer and stored at −80 °C. For the PEG precipitation [65], 80 µL of crude protein solution was suspended using 40% PEG solution for 30 s and then precipitated at −20 °C for 2 h. Further, the pellet was washed with 800 µL of cold acetone and desiccated, as described above. The final protein was resuspended in 80 µL of rehydration buffer (7 M urea, 2 M thiourea, 4% (*w*/*v*) CHAPS, 0.002% (*w*/*v*) bromophenol blue, 2% (*v*/*v*) Bio-Lyte, and 20 mM dithiothreitol). The concentration of the dissolved protein solution was determined according to the method of Bradford [66]. Then, the results were compared by routine SDS-PAGE test to optimize the method for protein extraction, and the extracted protein with the best quality was further separated by 2-DE for image analysis.

### 4.7. The 2-DE Assay and Image Analysis

Comparative 2-DE was performed using a Ettan^TM^ IPGphor apparatus (GE Healthcare, Pittsburgh, PA, USA) for isoelectric focusing (IEF; first dimension), and an Ettan^TM^ DALTsix (GE Healthcare) for the second dimension according to the manufacturer’s instructions [48] and the protocol described in a previous report [56]. For IEF, 300 µg hydrated protein was loaded on immobilized pH gradient (IPG) strips (18 cm length, pH 3.0–10.0). The following IEF steps were used: 500 V for 1 h, 1000 V for 1 h, 4000 V for 1 h, 8000 V for 1 h with a linear gradient, holding at 8000 V until a total of at least 40,000 Vh was reached, then holding at 500 V for 20 h. After the strips were balanced twice, the second dimension was performed with 12% SDS-PAGE gel, and the total proteins were stained before further analysis. The stained gel image was captured with an Image Master LabScan (GE Healthcare), and the images were analyzed using the ImageMaster^TM^ 2D Platinum 6.0 software (GE Healthcare) for spot detection, gel matching, and statistical analysis of the spots [56]. The image analysis remains one of the most labor-intensive parts of the 2-DE approach. In brief, triplicate images from three independent gels for the WT and mutant were obtained, while the normalization of the gels was carried out by the sum of the spot densities on each gel to compare the spots. The abundance of the individual protein spots was determined as vol.%. To identify the protein spots, the silver staining method was applied to the prepared gels. Silver nitrate was added to the solution before use, and then it was quickly admixed into the dyeing tray. The tray was then covered with an opaque cloth to reduce the decomposition of the silver nitrate utilized.

After visualization by staining and gel image analysis, protein spots with at least 3-fold spot volume ratio change (*p* < 0.05) were excised and subjected to mass spectrometry sequence analysis combined with database comparison. Statistical comparisons were conducted using the one-way ANOVA with the Tukey’s HSD test.

### 4.8. MALDI-TOF Analysis and Prediction of Differential Proteins

In this study, the spots showing statistically significant changes were cut out from the preparative gels and washed twice with ultrapure water. Then, the protein spots were destained with 50% acetonitrile (ACN) in 25 mM NH_4_HCO_3_. After removing the destaining buffer, the gel pieces were lyophilized and rehydrated in 30 μL of 50 mM NH_4_HCO_3_ containing 50 ng trypsin (Promega, Madison, WI, USA) at 37 °C overnight. The supernatant of the resulting peptides was washed with 0.1% trifluoroacetic acid (TFA) in 67% ACN. Extracts were pooled and lyophilized for MS analysis. The MS spectra were obtained using an ABI 4800 MALDI-TOF/MS-MS Proteomics Analyzer (Applied Biosystems, Foster City, CA, USA) as previously described [56]. The positive ion reflector (2 kV accelerating voltage) with 1000 laser shots per spectrum and automatic data acquisition modes were used for data collection, and the TOF spectra were collected over the mass range within 800–4000 Da with a signal-to-noise ratio minimum set to 10 and a local noise window width of *m/z* 250. A maximum of 10 precursors per spot with a minimum signal/noise ratio of 50 were selected for data-dependent MS/MS analysis.

Then, the resultant MS and MS/MS spectra data were analyzed with the GPS Explorer software (Version 2.0, Applied Biosystems). The database search was performed on the Mascot server (http://www.matrixscience.com) by searching the NCBInr (nonredundant protein sequence) database of *S. sclerotiorum* (http://www.ncbi.nlm.nih.gov/) to identify the proteins. The other important parameters were set as follows trypsin cleavage, two missed cleavage allowed, carbamidomethylation set as fixed modification, oxidation of methionine allowed as variable modification, monoisotopic precursor mass, precursor ion mass tolerance set to ±100 ppm, and fragment mass tolerance set to ±0.5 Da. The protein was correctly identified if a sufficient number of peptides were matched with a high score to a protein in the database. Only the significant hits with a protein score of 100% and the highest peptide counts were recorded and analyzed. Then the predicted protein sequences that matched the sequences in the NCBInr database were further analyzed for functional category denomination [56,62]. Prediction of the signal peptide was done using the online SignalP 4.1 Server (http://www.cbs.dtu.dk/services/SignalP/).

### 4.9. Data Analysis

All graphs were exported by the GraphPad Prism 6 software (La Jolla, CA, USA). Statistical comparisons were done using the one-way ANOVA with the Tukey’s HSD (Honestly Significant Difference) test in the PASW Statistics 18 (SPSS Inc., Chicago, IL, USA). 

## 5. Conclusions

In this study, we combined TCA/acetone precipitation for protein extraction, 2-DE, and peptide mass analysis to develop a fast and simple method for studying the proteomics changes of Δ*SsNsd1* mutant during compound appressorium formation. In our approach the results from 2-DE gel analysis are put into a larger context by combining spot data with functional annotations to explore the SsNsd1-mediated compound appressoria formation. Visualizing results, such as differential expression, functional categories, and predicted effector proteins makes it possible to gain new insights from the data accumulated by the “omics” technologies. Thus, this system approach can be effectively used to identify important candidate proteins in response to the SsNsd1-mediated appressorium formation, but it will require subsequent, more detailed studies to determine the precise role of the differentially expressed proteins.

## Figures and Tables

**Table 1:** List of the differential expression proteins identified as function known proteins*.*

Spot ^a^	Protein Name ^b^	Locus ^c^	Accession No. ^d^	Score ^e^	PM ^f^	Cov ^g^
01↓	nucleoside diphosphate kinase	gi/156063126	XP_001597486.1	360	6	44
03↓	nuclear transport factor 2	gi/156052963	XP_001592408.1	364	4	34
04↓	60 s ribosomal protein L23	gi/154703604	EDO03343.1	85	6	47
08↓	ubiquitin-conjugating enzyme E2	gi/156042748	XP_001587931.1	141	5	48
09↓	nucleoside diphosphate kinase	gi/156063126	XP_001597485.1	130	8	74
12↓	peptidyl-prolyl cis-trans isomerase	gi/156054872	XP_001593362.1	532	9	53
16↓	peptidyl-prolyl cis-trans isomerase B	gi/156042834	XP_001587974.1	583	10	37
17↓	nucleoside diphosphate kinase	gi/156063126	XP_001597485.1	281	5	39
18↓	eukaryotic translation initiation factor 5A-1	gi/156063512	XP_001597678.1	97	2	44
25↓	peptidyl-prolyl cis-trans isomerase	gi/156054872	XP_001593362.1	620	12	57
26↓	elongation factor 1-beta	gi/156053087	XP_001592470.1	107	2	31
28↓	60 s ribosomal protein L23	gi/154703604	EDO03343.1	59	2	62
29↑	peptidyl-prolyl cis-trans isomerase	gi/156054872	XP_001593362.1	406	8	46
30↓	GTP-binding nuclear protein GSP1/Ran	gi/156057585	XP_001594716.1	215	7	47
32-	SCF complex subunit Skp1	gi/156065065	XP_001598454.1	603	13	69
35↓	citrate synthase, mitochondrial precursor	gi/156063018	XP_001597431.1	104	9	42
39↓	peptidyl-prolyl cis-trans isomerase	gi/156054872	XP_001593362.1	498	11	51

^a^: The number of identified protein spots was showed on the two-dimensional gel electrophoresis (2-DE) image; The identified function known proteins were shown in this table; ^b^: Protein name searched by locus tag in NCBI result; ^c^: Locus tag number in NCBI annotation; ^d^: Accession number in NCBI; ^e^: Mascot score (threshold score > 50); ^f^: Peptide count; ^g^: Percent sequence coverage (%); ↓: expression level of proteins was down-regulated in the mutant; ↑: expression level of protein was up-regulated in the mutant; -: protein spot was disappeared in the mutant.

**Table 2:** List of the differential expression proteins identified as hypothetical proteins.

Spot ^a^	Protein Name ^b^	Locus ^c^	Accession No. ^d^	Score ^e^	PM ^f^	SignalP ^g^
02↓	hypothetical proteins	SS1G_05791	gi|156053886	412	6	No
05-	hypothetical proteins	SS1G_08534	gi|156049655	147	5	No
06↓	hypothetical proteins	SS1G_02967	gi|156061643	1090	16	Yes
10↓	hypothetical proteins	SS1G_13380	gi|156033320	329	7	No
11↓	hypothetical proteins	SS1G_10490	gi|156044772	136	5	No
14↑	hypothetical proteins	SS1G_05792	gi|156053888	404	6	No
15↓	hypothetical proteins	SS1G_03527	gi|156059030	58	3	No
19↓	hypothetical proteins	SS1G_03843	gi|156059662	545	10	No
20↓	hypothetical proteins	SS1G_06246	gi|156054796	143	4	No
21↓	hypothetical proteins	SS1G_06246	gi|156054796	658	10	No
22↓	hypothetical proteins	SS1G_11818	gi|156039363	1040	14	Yes
24↑	hypothetical proteins	SS1G_04923	gi|156055144	199	5	Yes
27-	hypothetical proteins	SS1G_09167	gi|156045782	46	7	No
31↓	hypothetical proteins	SS1G_02387	gi|156060497	237	5	No
33↓	hypothetical proteins	SS1G_06394	gi|156052457	389	4	No
34↓	hypothetical proteins	SS1G_02266	gi|156060255	1510	32	Yes
36↑	hypothetical proteins	SS1G_03994	gi|156056527	61	5	No
37↓	hypothetical proteins	SS1G_12818	gi|156036260	383	8	No
38↓	hypothetical proteins	SS1G_14285	gi|156031009	491	8	No
40↓	hypothetical proteins	SS1G_03527	gi|156059030	421	11	No
41↑	hypothetical proteins	SS1G_08260	gi|156049107	57	2	No
42↑	hypothetical proteins	SS1G_06959	gi|156051104	155	4	No
43-	hypothetical proteins	SS1G_06561	gi|156052787	186	3	No

^a^: The number of identified protein spots was showed on the 2-DE gel image; The hypothetical proteins were shown in this table; ^b^: Protein name searched by locus tag in NCBI result; ^c^: Locus tag number in NCBI annotation; ^d^: Accession number in NCBI; ^e^: Mascot score (threshold score > 50); ^f^: Peptide count; ^g^: Signal peptide predicted (Yes or No); ↓: expression level of protein was down-regulated in the mutant; ↑: expression level of protein was up-regulated in the mutant; -: protein spot was disappeared in the mutant.
